# Siderophore screening in marine sponge extracts using LC-HRMS and an R-based metabolomics workflow

**DOI:** 10.1371/journal.pone.0343544

**Published:** 2026-06-17

**Authors:** Alejandro García Ríos, Massuo Jorge Kato, Lydia Fumiko Yamaguchi, Breno Pannia Espósito, Ailan Farid Arenas-Soto

**Affiliations:** 1 Laboratorio de Química Bioinorgánica y Metalofármacos, Instituto de Química, Universidade de São Paulo, Sao Paulo, Sao Paulo, Brasil; 2 Grupo de investigación en Plaguicidas y Salud, Universidad del Quindío, Armenia, Quindio, Colombia; 3 Laboratório de Química de Produtos Naturais, Instituto de Química, Universidade de São Paulo, Sao Paulo, Sao Paulo, Brasil; 4 Grupo de Investigación y Asesoría en Estadística, Universidad del Quindío, Armenia, Quindio, Colombia; National Institute of Agricultural Research - INRA, MOROCCO

## Abstract

Siderophores are pivotal ‌‌iron-acquisition biomolecules integral to microbial survival, pathogenicity, and ecology. Elucidating these compounds offers critical insights into the microbial dynamics of marine holobionts and potential therapeutic applications. In this study, we present a culture-independent, data-centric strategy to annotate siderophores from the body mass of three marine sponge species: *Dragmacidon reticulatum*, *Aplysina fulva*, and *Amphimedon viridis*. Utilizing Liquid Chromatography-High Resolution Mass Spectrometry (LC-HRMS) coupled with a custom R-based analytical workflow (XCMS and MetaboAnnotation), we putatively annotated 59 siderophores. We employed a validation pipeline, utilizing iron-adduct calculations [M-2H + Fe]^+^, [M-H + Fe]^2+^, [2M-2H + Fe]^+^, mass accuracy thresholds (<3 ppm), retention time deviation (Coefficient of variation < 2%), and chromatograph peak analysis. According to the Metabolomics Standards Initiative (MSI), these annotations correspond to Level 2 (putatively annotated compounds) because they are based on accurate mass matching without chemical standard confirmation. Notably, iron supplementation during extraction did not significantly alter siderophore detection, suggesting constitutive production or environmental saturation. This workflow bypasses the limitations of traditional cultivation, revealing a diverse landscape of iron-chelating metabolites, including Ferricrocin, Aeruginic acid, and Madurastatin directly within the sponge’s body.

## Introduction

Siderophores are ‌‌small, high-affinity iron-chelating compounds produced by microorganisms, including bacteria and fungi. These molecules play a crucial role in microbial survival and pathogenicity by facilitating iron acquisition from the environment [1–2]. Although iron is an essential nutrient for nearly all living organisms, its bioavailability is limited under aerobic conditions because of its low solubility. Siderophores overcome this limitation by binding iron with high specificity and affinity, thereby enabling its uptake into microbial cells [1–2].

In a health context, siderophores are of considerable interest due to their roles in both pathogenesis and clinical applications. In many pathogenic bacteria, siderophores are virulence factors by enabling efficient iron sequestration in the host’s iron-limited environments, thereby promoting infection and disease progression [3]. Beyond their role in virulence, siderophores exhibit significant therapeutic potential. Their strong iron-chelating properties have been explored for the treatment of iron overload disorders, such as hemochromatosis and certain anemias [4–5]. In addition, synthetic siderophores and siderophore–drug conjugates have been developed to deliver antibiotics selectively into bacterial cells, enhancing treatment efficacy while reducing host toxicity [6].

Siderophores also hold promise for diagnostic applications. Their distinctive biochemical properties can be leveraged to develop sensitive and specific tools for detecting bacterial infections, particularly those caused by siderophore-producing pathogens [7].

Several methodologies have been developed for the detection, identification, and quantification of siderophores, each with distinct strengths and limitations [8]. The Chrome Azurol S (CAS) assay is a widely used colorimetric method that detects siderophore-mediated iron chelation through a characteristic color change, providing a simple and rapid screening tool for siderophore production. However, the CAS assay is non-specific, as it cannot distinguish between different siderophore types or identify individual structures, and it is subject to interference from other iron-chelating compounds in complex matrices [8–10]. Bioassays employing siderophore-utilizing indicator strains show bioactivity information and reveal functional siderophore diversity. These approaches are inherently cultivation-dependent and low-throughput, limiting their applicability to environmental samples with unculturable microbiomes [10–11]. Nuclear magnetic resonance (NMR) spectroscopy provides definitive structural elucidation of purified siderophores; however, NMR requires pure compounds in milligram quantities and is impractical for analyzing complex environmental extracts, where siderophores occur at low concentrations amid abundant co-extracted metabolites [8–12]. Liquid chromatography coupled with tandem mass spectrometry (LC-MS/MS) and molecular networking platforms such as the Global Natural Products Social Molecular Networking (GNPS) enable structural annotation through fragmentation pattern analysis and spectral library matching. These approaches depend heavily on the availability of well-curated MS/MS spectral libraries, which remain limited for many siderophore classes [13].

Liquid chromatography coupled with high-resolution mass spectrometry (LC-HRMS) has emerged as a powerful analytical approach for the identification and quantification of siderophores. By integrating the separation efficiency of liquid chromatography with the analytical performance of high-resolution mass spectrometry, LC-HRMS provides a sensitive, specific, and high-throughput platform for detecting siderophores, even within complex biological matrices [13–14]. Compared to the methods described above, LC-HRMS with accurate mass matching offers a complementary balance of sensitivity, throughput, and broad coverage for initial screening of complex environmental samples, while acknowledging that it does not replace definitive structural confirmation by NMR or MS/MS-based approaches [13–14]. Despite LC-HRMS techniques and protocols, some pitfalls need to be overcome when identifying siderophores. They often occur in low concentrations, especially in complex environments. Their low abundance compared to other metabolites requires sensitive analytical methods for detection. Siderophores have diverse chemical structures that can vary significantly among different organisms. This structural diversity requires comprehensive databases and sophisticated analytical techniques to accurately identify and differentiate siderophores from other molecules [15].

The acquisition, processing, and management of high-resolution spectral data from complex samples are critical for the identification of siderophores. This process relies on computational tools and statistical methods implemented through established algorithms in specialized software and databases, such as XCMS [16]. These resources support the development of workflows for analyzing high-resolution mass spectrometry datasets, which typically comprise gigabytes of data. The datasets are structured and analyzed using parameters such as retention time, intensity, and m/z, often through freely accessible platforms such as R.

There is a growing demand for cost-effective methodologies for detecting siderophore production in complex environmental samples. In parallel, the limited accessibility of curated siderophore databases hampers accurate compound identification [17]. Consequently, accurate siderophore identification depends on efficient computational algorithms able to process large datasets and discriminate siderophores from closely related compounds [18].

Symbiotic microorganisms associated with marine sponges, particularly bacteria, represent a rich source of natural products with diverse biological activities, including novel siderophores that remain unexplored in other environments [19]. In this study, we applied an R-based workflow for siderophore annotation utilizing high-resolution mass spectrometry data obtained from the body tissue of the marine sponges *Dragmacidon reticulatum*, *Aplysina fulva*, and *Amphimedon viridis* to expand siderophore annotation in marine environments. This workflow integrates established R packages, including XCMS, MetaboAnnotation, and ggplot2, based on exact mass matching against the current version of the SIDERITE database [20–21].

## Materials and methods

### Samples

Following methanol extraction of the 18 samples, the resulting extracts of 250 μL were split into two aliquots of 98 μL: one supplemented with 2 μL of 200 μmol L ⁻ ¹ Ferrous Ammonium Sulfate (FAS) (final concentration 4 μmol L ⁻ ¹) as an iron source to promote the formation of iron adducts [22]; the control aliquot was supplemented with 2 μL of ultrapure water. This yielded a total of 36 samples for LC-HRMS injection.

### Sample preparation

For sample preparation, each 2 g of unfrozen sponge tissue was weighed into 50 mL Falcon tubes and subjected to three successive ethanol extractions (15 mL per extraction), each involving 1 min of vortexing followed by 3 min of sonication at 40 kHz. After centrifugation at 1400 rcf, the supernatants were combined and stored at 4 °C. The resulting ethanolic extract was concentrated using a Buchi Rotavapor R215 with a recirculating chiller (F100) and heating bath (B491) under vacuum (0.074 atm) at 35 °C and 130 rpm, keeping the distillation head below 24 °C. Approximately 100 mg of dried extract for each sample was then redissolved in 5 mL of water, thoroughly vortexed, and partitioned with chloroform in four successive 5 mL portions. Each mixture was vortexed for 2 min at 3000 rpm, allowed to equilibrate at 4 °C for 15 min, and centrifuged at 400 rcf for 3 min to separate the aqueous and organic phases. The organic fraction was dried in an Eppendorf SpeedVac Plus (Volatile Heat Mode, no heating) for 5 h. 20 mg of dry organic fraction was redissolved in 5 mL of methanol, and a 500 µL aliquot was filtered through a pre-conditioned Millipore Ultracel PL-10 centrifugal device at 14000 rcf and 4 °C for 20 min. The filtrate was dried again in the SpeedVac and finally redissolved in 250 µL of dry methanol for LC-HRMS analysis, obtaining a final concentration of the organic extract of 5 mg mL^-1^ for each sample. All steps were performed under controlled temperatures to preserve thermo-labile compounds, providing an efficient and reproducible workflow for the extraction, fractionation, and preparation of sponge-derived metabolites for high-resolution mass spectrometry.

### Analysis by RPLC – HRMS

Liquid chromatography analyses were performed on a Shimadzu LC coupled to electrochemical ionization in an online quadrupole detector with a time-of-flight detector (qToF Bruker Daltonics). The samples of each ultrafiltered organic fraction in methanol (250 µL) were divided into two vials, with 98 µL each. Then, the first vial was added with 2 µL of FAS, 200 µmol L^-1^ (with Fe), and the second, with 2 µL of Milli-Q water (without Fe). An aliquot of 5 µL of the organic fraction with and without iron was injected into column C18 (150 × 3 mm DI, 5 m) Ascentis® express (Sigma-Aldrich) with a precolumn. The conditions of the method were water (solvent A) and methanol (solvent B) as mobile phase (both with 0.1% formic acid, v/v) at 0.4 mL min^-1^ and 40°C. The chromatographic conditions were 0–10 min (20–100%B), 10–20 min (100%B), 20–23 min (20%B), 23–30 min (20%B). All samples (with and without iron addition) were analyzed by LC-qToF with the mass band, m/z from 150 to 2000 Da, at a speed of 1 spectrum s^-1^. The voltage in the capillar was 3500 V; in the fragmenter, 125 V; in the skimmer, 65 V; in the Nozzle, 1000 V, and in the octapole, 750 V. Into the ESI, the atomizer conditions were 30 psi at 250°C and 8 L min^-1^ argon.

### Data processing XCMS R Package

36 raw LC-HRMS signals were converted to the standard mzXML format using msConvert from the ProteoWizard tool suite. The converted mzXML files range from 0.3 GB per sample. The 36 mzXML files were preprocessed for chromatogram alignment, noise removal, feature detection, and integration. The mass features were extracted using the XCMS 4.0.2 R package standard protocol (https://bioconductor.org/packages/release/bioc/html/xcms.html). We applied 5 centWave peak detection methods, varying the arguments to capture the more specific siderophores in the samples [23] (S1 Text). The overall centWave peak detection function was defined between 3 and 60 seconds. Resolution in parts per million (ppm) between 5 and 10, specifying an m/z difference (mzdiff) of 0.001–0.005. Further preprocessing involved prefilters with thresholds ranging from 2 to 2000, and noise minimization was capped at 10000. The missing values were placed using the fillChromPeaks function [16,20]. The final metabolite features table was in a “csv’‘ format file.

### Siderophores dataset

Siderophore information for exact mass and hosted microorganisms was obtained from the latest version of the SIDERITE database (version 01-20-2025). This dataset contains information on 984 unique siderophore structures sourced and curated from the scientific literature [21].

### Adducts calculated

Previously, we calculated for each “exact mass” in the SIDERITE dataset the adduct [M + H]+ by adding the mass of a hydrogen atom. Similarly, an R routine was run to obtain the more plausible iron adducts from each siderophore’s “exact mass”. The iron Adducts were as follows: “[M-2H+Fe]+”, “[M-H+Fe]2+” and “[2M-2H+Fe]+”.

### M/Z matching approach with metaboAnnotation R package

The m/z matching approach consisted of accurately matching the m/z obtained in the metabolite feature table directly against the m/z adducts calculated from the SIDERITE database using the Mass2MzParam and MatchValues functions from the MetaboAnnotation R package 1.6.1 [24], with a predefined tolerance of 0.005 and ppm < 3.

### Data analysis

The information for all m/z features that matched siderophores was analyzed using R code routines using the ggplot2 library.

### Chromatographic peak analysis

Finally, we identified the specific chromatographs that matched the m/z detected in the feature table. From the raw mass spectrometry mzXML files and the m/z obtained in the feature table, we plotted all the chromatogram peaks for all putative siderophores using the function “chromatogram” from the XCMS R package and standard plot drawing functions. This visual inspection of the peaks serves as a criterion to annotate the target siderophore. The bandwidth for each m/z was (+/- 0.001), and a retention time of 60 seconds was chosen [23].

## Results

**Fig 1:**
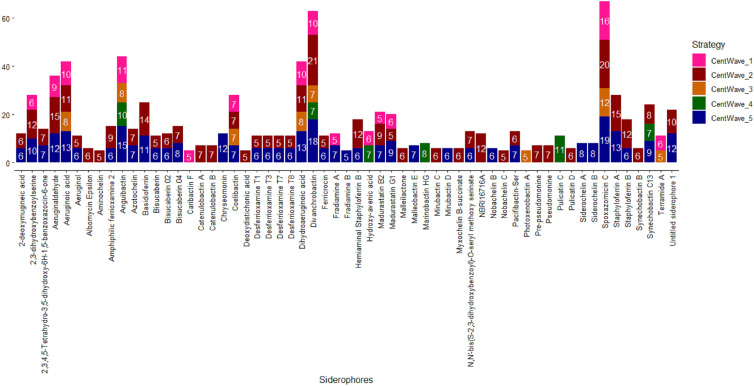
Putative annotated Siderophores matched in the Siderite dataset. Number of putative siderophores found for each centWave function compound detection. It showed siderophores with 5 or more m/z features and ppm < 3.

**Fig 2:**
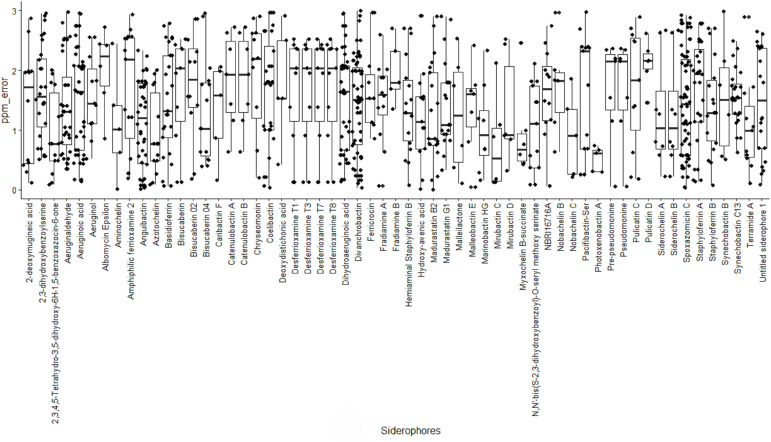
Parts-per-million (ppm) error distribution for each putative Siderophore. Boxplots illustrate the (ppm) error distribution for all 59 annotated putative siderophores.

Initially, 62283 peaks were obtained in the feature table after applying the XCMS protocol to the 36 raw mzXML signal files. These m/z were matched against the adducted siderophores in the SIDERITE dataset using the MetaboAnnotation R package. Applying 5 “centWave” functions (by modifying their arguments) yielded 59 putative siderophores ‌‌(Figs 1 and 2). All the putative siderophores found were adjusted to less than 3 ppm error and more than 5 m/z matches per siderophore (Figs 1 and 2). Fig 1 highlights that different strategies can capture a full metabolomic profile. While Strategy 5 (dark blue bars) was highly effective for detecting compounds Divanchrobactin and Spoxazomicin C, CentWave_1 (pink bars) was necessary to capture specific isomers of Aeruginaldehyde. This multi-tiered approach yielded 59 distinct putatively annotated siderophores, significantly expanding coverage compared to standard single-parameter processing.

### Distribution of iron adduct-forming chelating siderophore

**Fig 3:**
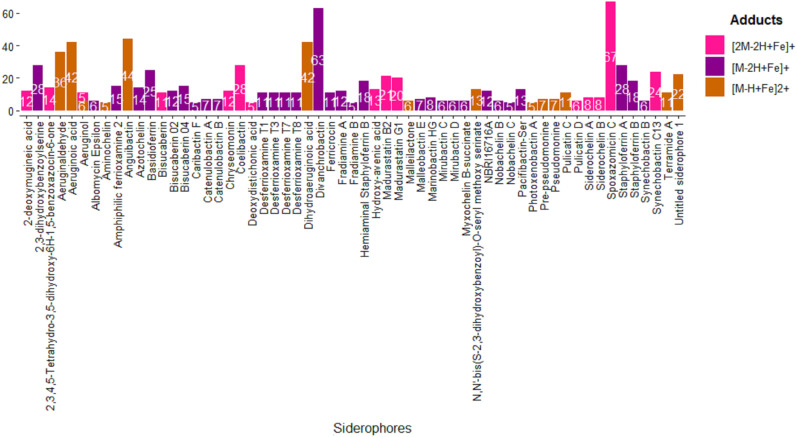
Distribution of (Fe^3+^) ionization adduct-forming. The bar chart illustrates the m/z detected for 59 putative adduct-forming siderophores. Each bar reflects the number of features for each siderophore.

We further analyzed the ionization behavior of these compounds. Fig 3 reveals that the singly charged iron adduct [M-2H + Fe]^+^ is the dominant species, accounting for the identification of 31 unique siderophores. This is consistent with the coordination chemistry of hexadentate siderophores, which typically lose two protons to coordinate a ferric ion (Fe^3+^), resulting in a net + 1 charge. However, the presence of [M-H + Fe]^2+^ (orange bars) and dimer adducts (pink bars) of Aeruginic acid indicates diverse ionization pathways that must be accounted for in untargeted screening (Fig 3) [25].

### Coefficient of variation (CV) of the retention time for all m/z values of each siderophore

**Fig 4:**
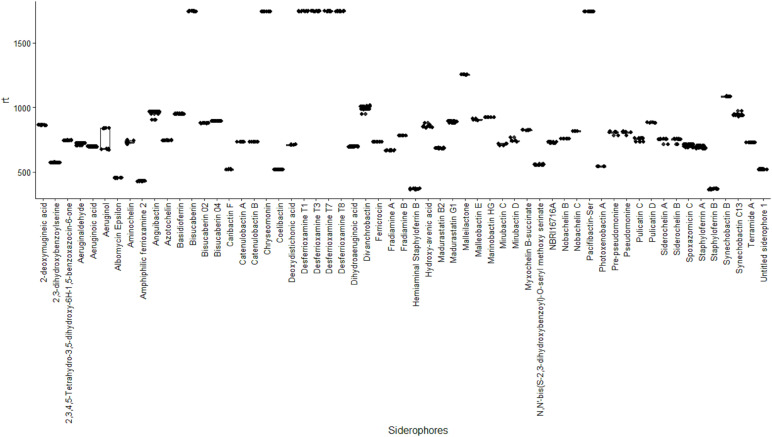
Coefficient of variation (CV) of the retention time (rt) for each m/z corresponding to a specific siderophore. Each short horizontal line represents the rt variation for a given putative siderophore. Only siderophores with CVs below 2% are shown.

To rule out false positives arising from noise, we assessed retention time (RT) variation as a key criterion for annotating siderophores. The coefficient of variation (CV) provides a standardized measure of RT dispersion for each m/z corresponding to a specific siderophore. Lower CV values indicate tighter clustering of RTs, reflecting greater consistency in the chromatographic behavior of the associated m/z. Our results showed that all 59 putative siderophores exhibited RT dispersions below 2% (Fig 4), suggesting the reliability of their detection.

### Effects of iron supplementation on the detection of Siderophore masses

**Fig 5:**
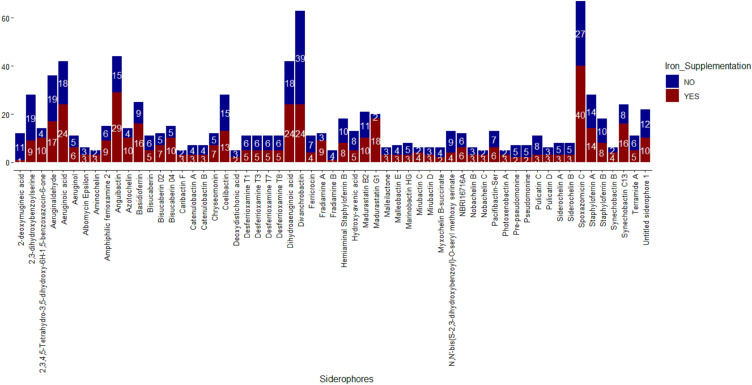
Comparing iron supplementation and non-supplementation on the siderophore m/z detected. The bar plot illustrates differences in m/z for each siderophore when iron was supplemented (“YES”) versus when it was not (“NO”).

Interestingly, in vitro iron supplementation did not significantly enhance siderophore annotation (Fig 5). The counts of detected features were statistically indistinguishable between iron-supplemented (YES) and non-supplemented (NO) samples. This suggests that the annotated siderophores in these sponge extracts were already present as iron complexes (ferri-siderophores) due to the ambient iron in the sponge tissue, or that their production is constitutive rather than strictly inducible under these extraction conditions (Fig 5).

### Chromatogram profiles for each detected Siderophore

**Fig 6:**
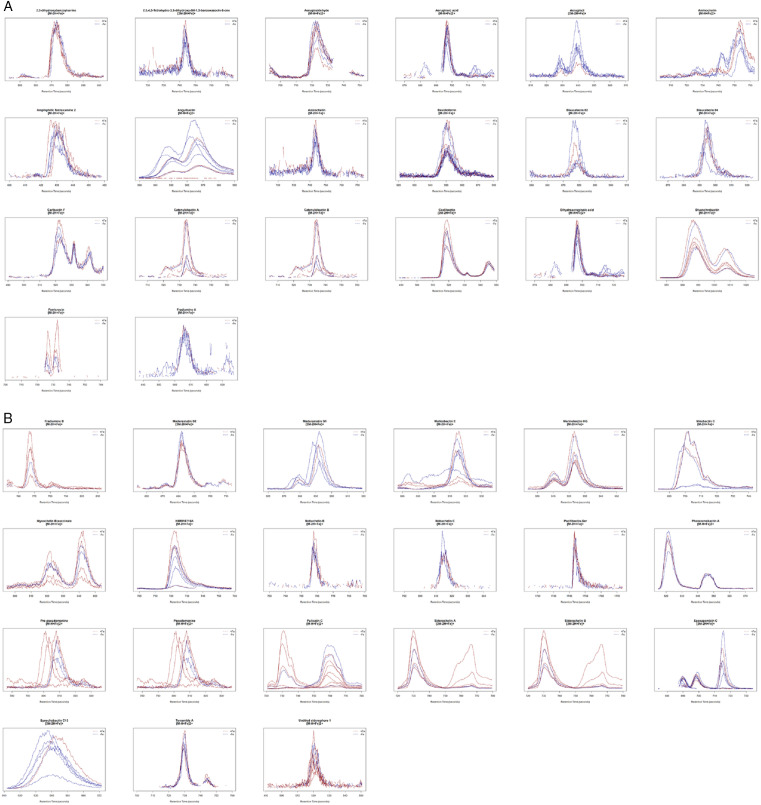
Chromatograms for the more probable siderophores annotated in the sponge samples. The bandwidth for all images was set to +/-0.001 m/z for each siderophore, and a retention time of 1 min is shown.

The final test of our workflow is evidenced by the extracted ion chromatograms (EICs) for each putative siderophore. Fig 6A and 6B display the elution profiles of the 41 most confident identifications. Most of the chromatograms exhibit sharp Gaussian peak shapes (e.g., Madurastatin G1 and Nobachelin B), indicative of successful separation from the complex sponge matrix. For many compounds, such as Aeruginaldehyde ‌‌(Fig 6A), we observe co-elution or slight retention time shifts between the Fe-supplemented (red lines) and non-supplemented (blue lines) traces. This suggests the presence of apo and ferri-forms dynamically equilibrating within the column or source. Notably, Fig 6B shows distinct peaks for structural variants, such as the Madurastatin series (B2, G1) and Nobachelin variants. The ability to resolve Madurastatin G1 (RT ~ 890s) from Madurastatin B2 (RT ~ 680s) demonstrates that the chromatographic method is well-suited.

## Discussion

This study reports the putative annotation of siderophores—crucial iron-chelating compounds—from microorganisms symbiotically associated with marine sponges. We used a methodology that combines Liquid Chromatography-High Resolution Mass Spectrometry (LC-HRMS) with data processing and analytical tools in R, including the XCMS [16,20], MetaboAnnotation [24], and ggplot2 packages. The combination of these analytical tools provides a thorough and efficient workflow for metabolite discovery from mass spectrometry data. This approach implements a mass-feature matching protocol against a curated and highly specialized siderophore database [21], which resulted in the putative annotation of 41 siderophores directly obtained from the body tissue of three distinct sponge species, all accomplished without the necessity for the prior isolation or cultivation of the associated microorganisms (Figs 1–5).

We applied a filtration process based on diverse criteria: the calculation of three distinct iron adducts, a parts-per-million (ppm) resolution threshold of less than 3 for all feature adducts, m/z feature counts per siderophore greater than 4, a coefficient of variation in the retention time that does not exceed 2% across all m/z features associated with each annotated siderophore (Figs 2–4), and the plotting of the chromatograms (Fig 6A and 6B). Implementing these criteria enhances the reliability of our annotation process for these specialized chemical compounds (S2 Text). However, it is important to note that, according to the Metabolomics Standards Initiative (MSI), our annotations correspond to Level 2 (putatively annotated compounds), as identification relies solely on accurate mass matching without confirmation by authentic chemical standards [26]. Future studies incorporating tandem MS (MS/MS) fragmentation patterns and comparison with authentic standards would be necessary to achieve Level 1 (confirmed) identification.

Several tools are available for metabolite identification from LC-MS experiments, including MetaboAnalyst [27], MS-DIAL [28], MZmine [29], OpenMS [30], MetFrag [31], GNPS [13], and MetaboLights [32]. Programmatic approaches using languages such as R offer distinct advantages in terms of flexibility and adaptability to various analytical scenarios. In addition, the ggplot2 package enhances data visualization, enabling the creation of customized graphics tailored to specific analytical requirements, which improves the interpretability and presentation of results from complex datasets (Figs 1–5).

Siderophores remain underexplored in various environmental and biological matrices. Previous studies have typically involved isolating and culturing microorganisms before LC-MS analysis. Mawji et al. (2011) detected 22 siderophores from Atlantic Ocean waters enriched with various carbon and nitrogen substrates, enhancing siderophore capture [33]. Lehner et al. (2013) used isotope-assisted screening to identify 18 iron chelators produced by 10 wild-type strains of the Trichoderma genus under laboratory cultivation [34]. Baars et al. (2015) identified 35 distinct siderophores produced by the nitrogen-fixing soil bacterium Azotobacter vinelandii, which is widely recognized for its significant role in the nitrogen cycle [35]. Boiteau et al. (2019) reported 9 siderophores from cultured soil microorganisms, including Schizokinen, which was also found in our screening [36]. Liu et al. (2022) reported 10 siderophores isolated from the actinomycete Streptomyces diastaticus NBU2966, associated with a marine sponge of the Axinellida order [37]. Our study represents one of the few instances in the scientific literature that investigates the presence of siderophores produced by microorganisms inhabiting the ecological niche provided by sponges [38]. Our study contributes to this field and enhances the existing body of knowledge about the presence and relevance of siderophores in marine environments, specifically associated with sponges.

The ecological significance of the putatively annotated siderophores in this study warrants particular attention. Iron is a limiting nutrient in oligotrophic marine environments, and the competition for this essential metal among microbial communities is intense [39]. Siderophore-producing bacteria may play a critical role in maintaining iron homeostasis, benefiting both the microbial community and the host organism [40]. For instance, Ferricrocin, one of the most frequently putatively annotated siderophores in our study, is produced by diverse fungi and bacteria and serves as an intracellular iron storage molecule, helping to regulate intracellular iron concentrations and prevent oxidative damage [41]. Aeruginic acid, another compound putatively annotated in our samples, has been associated with Pseudomonas species and may contribute to competitive interactions within the microbial community by sequestering iron from neighboring organisms [42]. Madurastatin-type siderophores, originally isolated from Actinomadura species, possess unique structural features that suggest specialized ecological roles, potentially influencing microbial community structure through iron-mediated competition [43]. The production of diverse siderophores by sponge-associated microorganisms may serve as an ecological mechanism to maintain symbiotic relationships, while the host sponge benefits from iron acquisition facilitated by microbial metabolites [40,44]. This mutualistic exchange underscores the importance of siderophores as chemical mediators of symbiosis in marine holobionts.

The literature concerning the identification of siderophores synthesized by sponge-associated microbial communities is quite scarce. A particular investigation revealed that microorganisms extracted from sponges exhibited no detectable siderophore production, even when iron was supplemented to the culture medium [45]. Nevertheless, certain isolated bacterial strains demonstrated the capability to produce siderophores when stimulated by exogenous siderophores, suggesting a potential signaling function of exogenous siderophores in enhancing the production of indigenous siderophores in marine bacterial populations [45]. In our experimental methodology, we noticed that *in vitro* iron stimulation did not result in a significant difference in the count of m/z siderophores (Fig 5). We suspect that most of the putative annotated siderophores are strong iron-chelator compounds, and probably they were already forming chelating complexes, such that no increasing chelation activity was observed with exogenous iron supplementation (Fig 5).

Based on the 41 distinct siderophores putatively annotated in this investigation, we propose that sponges represent promising reservoirs for siderophores within their native ecosystems, particularly given the prevalent iron scarcity in seawater and the intense competition for this vital metal [39]. The symbiotic interaction between sponges and their associated microorganisms may be fundamentally influenced by the distinctive ecological niches present within the sponge species [46]. The diverse siderophore repertoire here suggests that iron-mediated chemical interactions are an important component of the sponge–microbe symbiosis, with implications for holobiont health and ecological resilience.

## Conclusions

Our study employed a homemade, data-driven approach combining Liquid Chromatography-High Resolution Mass Spectrometry (LC-HRMS) with data processing in R using packages such as XCMS, MetaboAnnotation, and ggplot2 to annotate putative siderophores from sponge samples. This methodology detected 41 distinct siderophores across three sponge species: *Dragmacidon reticulatum*, *Aplysina fulva*, and *Amphimedon viridis*, without prior isolation or cultivation of associated microorganisms. These annotations correspond to MSI Level 2 (putatively annotated compounds), as they are based on accurate mass matching without confirmation by authentic chemical standards. This research highlights the potential of marine sponges as reservoirs for diverse siderophores, which could serve as indicators of marine environmental quality and ecosystem health. *In vitro* iron stimulation did not significantly alter siderophore annotation. This research contributes to the limited literature on siderophores associated with marine sponges.

## Supporting information

S1 Text Contains the centWave R functions with the arguments used in this study. (TXT)

S2 Text Contains the R scripts for the basic siderophore annotation. (DOCX)
