# Supplementary material for: Siderophore screening in marine sponge extracts using LC-HRMS and an R-based metabolomics workflow
Source: PLoS One. 2026 Jun 17;21(6):e0343544. doi: 10.1371/journal.pone.0343544 (PMC13274820; doi:10.1371/journal.pone.0343544)
Supplement: S2 Text — (DOCX) [file pone.0343544.s002.docx]

**Supporting Information S2.**

- ***36 mzXML files were processed with XCMS R Package.***

setwd("E:/") “set work directory with all files available”

mzXML <- list.files(pattern = ".mzXML", recursive = TRUE)

library(c(“xcms”, “MSnbase”))

raw_data <- readMSData(files = mzXML, pdata = new("NAnnotatedDataFrame", pd), mode = "onDisk")

*# pd is a dataframe with 36 rows with the names of the 36 samples and all variables associated with each sample in columns” (columns are: iron supplementation, season, sponge specie, etc)*

- ***Retention time filtration, alignment, and Peak detection***

raw_data <- filterRt(raw_data, c(0, 1300))

cwp<- CentWaveParam(ppm = 5, peakwidth = c(5, 20), snthresh = 10, prefilter = c(3, 1000), mzCenterFun = "wMean", integrate = 1, mzdiff = 0.001, noise = 500

xdata <- findChromPeaks(raw_data, param = cwp)

Features<-chromPeaks(xdata)

write.csv(Features, "Features.csv")

# Supplementary Material S1 contains the five centWaveParam methods (modifying the arguments)

***Adducts Calculated*.**

library(MetaboAnnotation)

adducts=MetaboCoreUtils::adductNames(polarity = "positive")

No-iron-adducts:

"[M+H]+"

Iron Adducts:

“[M-2H+Fe]+”, “[M-H+Fe]2+” and [2M-2H+Fe]+.

***Calculating iron adducts example: [M-2H+Fe]+***

#M is the column of the "exact masses" for each siderophore in the SIDERITE #database [15].

H=1.007825 # hydrogen

Fe=55.934939 # iron

x=M

x1=2*H # 2H

x2=x-x1 # M-2H

x3=x2+Fe # M-2H+Fe

# Similar calculations were done for each iron adduct.

# vectors for each iron adduct are calculated from SIDERITE exact mass column.

# You must save the column into a dataframe, for example, we called “add_iron”.

- ***Match molecular weights between the feature table vs iron adduct table with the MetaboAnnotation R Package.***

parameters <- Mass2MzParam(adducts = adducts, tolerance = 0.005, ppm = 0)

matched_features <- matchValues(Features[,1], add_iron[1], parameters)

matched_features <- matchedData(matched_features)

matched_features <-as.data.frame(matched_features)

matched_features <- matched_features[complete.cases(matched_features)

#Features is the dataframe with all peaks obtained in the processing. Be sure to #designate the column with the m/z values Features[,1].

#add_iron is the dataframe with a column with the particular iron adduct calculated.

#this must be run three times or integrated into a cycle to match each iron adduct #add_iron[1:3]
