# Supplementary material for: Siderophore screening in marine sponge extracts using LC-HRMS and an R-based metabolomics workflow
Source: PLoS One. 2026 Jun 17;21(6):e0343544. doi: 10.1371/journal.pone.0343544 (PMC13274820; doi:10.1371/journal.pone.0343544)
Supplement: Attachment — Submitted filename: Response to Reviewers.pdf [file pone.0343544.s005.pdf]

Dears Editor and reviewers,

On behalf of all authors, we would like to thank you and the reviewers for the constructive feedback and the opportunity to revise our manuscript "Siderophore identification in microorganisms associated with marine sponges by LC-HRMS and a data analytic approach in R" (PONE-S-26-08981).

Below, we provide a point-by-point response to the reviewer's comments.

- **Reviewer Comment:** The title is misleading as the study analyzes sponge extracts, not isolated microorganisms. Suggests: *"Siderophore screening in marine sponge extracts..."*

Response: We acknowledge the reviewer's concern regarding the biological source. Our study utilizes a metabolomics approach, aiming to capture the Siderophore landscape of the sponge holobiont (the host and its complex microbial community). To resolve any ambiguity, we change the title to: **"Siderophore screening in marine sponge extracts using LC-HRMS and an R-based metabolomics workflow."**

- **Reviewer Comment:** In the introduction section, the authors should have discussed other methodologies that are used to identify and quantify the siderophores. The strengths and weaknesses of such methods should have been highlighted.

Response: We added the two paragraphs below in the introduction section regarding other methods to identify Siderophores.

Several methodologies have been developed for the detection, identification, and quantification of siderophores, each with distinct strengths and limitations. The Chrome Azurol S (CAS) assay is a widely used colorimetric method that detects siderophore-mediated iron chelation through a characteristic color change, providing a simple and rapid screening tool for siderophore production. However, the CAS assay is non-specific, as it cannot distinguish between different siderophore types or identify individual structures, and it is subject to interference from other iron-chelating compounds in complex matrices. Bioassays employing siderophore-utilizing indicator strains offer biological activity information and can reveal functional siderophore diversity, yet these approaches are inherently cultivation-dependent and low-throughput, limiting their applicability to environmental samples with unculturable microbiomes. Nuclear magnetic resonance (NMR) spectroscopy provides definitive structural elucidation of purified siderophores; however, NMR requires pure compounds in milligram quantities and is impractical for the analysis of complex environmental extracts where siderophores occur at low concentrations amid abundant co-extracted metabolites. Liquid chromatography coupled with tandem mass spectrometry (LC-MS/MS) and molecular networking platforms such as the Global Natural Products Social Molecular Networking (GNPS) enable structural

annotation through fragmentation pattern analysis and spectral library matching, but these approaches depend heavily on the availability of well-curated MS/MS spectral libraries, which remain limited for many siderophore classes.

"*Compared to the methods described above, LC-HRMS with accurate mass matching offers a complementary balance of sensitivity, throughput, and broad coverage for initial screening of complex environmental samples, while acknowledging that it does not replace definitive structural confirmation by NMR or MS/MS-based approaches.*"

- **Reviewer Comment**: Several statements have not been supported with literature (the authors should provide relevant citations for these statements).

Response: We carefully reviewed the entire introduction section to ensure that relevant citations properly support all statements. In the revised manuscript, we added new references for any uncited claims.

- **Reviewer Comment:** Unclear sample collection and experimental Design.

Response: We clarified in the revised text all issues regarding sample acquisition and the inconsistency in the methodology. The following paragraph was replaced in the methodology section "Samples".

"*Samples were collected from three sponge species: D. reticulatum, A. fulva, and A. viridis, along the São Paulo coast, Brazil, at depths of 3–8 m, by SCUBA diving (ICMBio permit #0289170220250814). Three independent individuals per species were sampled as biological replicates. From each individual, two separate 2-g portions of body tissue were excised from two different anatomical regions, serving as technical replicates, for a total of 18 samples (3 species × 3 individuals × 2 tissue portions). Immediately after collection, samples were flash-frozen in liquid nitrogen on board, transported on dry ice, and stored at −80 °C until extraction.*

Following methanol extraction of the 18 samples, the resulting extracts were each split into two aliquots: one supplemented with 200 µmol L^{-1} Ferrous Ammonium Sulfate (FAS) (final concentration 4 µmol L^{-1}) as an iron source to promote the formation of *iron adducts [22]; the control aliquot was supplemented with 2* µL of *ultrapure water. This yielded a total of 36 samples for LC-HRMS injection."*

- **Reviewer Comment:** Why was 200 µmol/L used? Why Iron(III) Ammonium Citrate (FAS)?

Response: First, we corrected the substance used: *Iron(III) ammonium citrate (FAS), which is not the compound used in the chelation stimulation protocol. The substance used was* Ferrous Ammonium Sulfate (FAS). The concentration of 200 µmol/L was selected based on established siderophore induction and detection *in-house* protocols in our lab, providing sufficient iron to saturate available ligands

without inducing toxicity or massive precipitation that could interfere with LC-HRMS analysis. Ferrous Ammonium Sulfate (FAS) was chosen for its high solubility in both water and organic mixtures, ensuring consistent iron availability. The fact that supplementation did not significantly alter detection suggests that the identified siderophores were already present as iron-complexes (ferri-siderophores) or were constitutively produced within the sponge environment. In this reference, *"Batko, I. Z., Flannagan, R. S., Guariglia-Oropeza, V., Sheldon, J. R., & Heinrichs, D. E. (2021). Heme-Dependent Siderophore Utilization Promotes Iron-Restricted Growth of the Staphylococcus aureus hemB Small-Colony Variant. Journal of bacteriology, 203(24), e0045821. https://doi.org/10.1128/JB.00458-21"* [22]. The authors use a concentration of 10 µM of FAS to complement the growth of a *Staphylococcus aureus* hemB Small-Colony Variant, which is a bacterium defective in growing in media with iron deficiency. Adding FAS restores bacterial growth. The final concentration of FAS used in our experiment was 4 µmol L^{-1} since we added 2 µL of 200 µmol L^{-1} Ferrous Ammonium Sulfate (FAS) to 98 µL of extract before LC-HRMS injection. All those quantities and concentrations were clarified in the revised manuscript.

- **Reviewer Comment**: The mass of dried extract obtained is not reported, and the final concentration of the extract before LC-MS injection is unclear.

Response: We acknowledge that this information is important for analytical reproducibility and will add it to the revised manuscript. While we did not record individual dry weights for each of the 18 biological samples (as this was not a primary objective of the study), we can provide the following details: the final reconstitution volume was 250 µL of dry methanol, and the injection volume for LC-HRMS was 5 µL per run. Based on our experience with similar marine sponge ethanol extracts processed through the same fractionation workflow, the typical dry extract yield from 2 g of wet sponge tissue after chloroform partition is in the range of 5–20 mg, corresponding to a final concentration of approximately 5 mg/mL in 250 µL methanol. We will add these estimates to the revised manuscript. We will also note that the injection of 5 µL at these concentrations is consistent with standard LC-HRMS practices for natural product screening.

- **Reviewer Comment:** Claims of identifying specific compounds (Ferricrocin, etc.) are overinterpreted without MS/MS or standards.

Response: We agree that in untargeted metabolomics, these represent putative identifications (Level 2/3 according to the Metabolomics Standards Initiative). However, we defend our current results based on our multi-orthogonal validation pipeline:

Mass Accuracy: All reported features had a mass error ppm < 3.

Adduct Matching: We didn't rely on a single mass; we calculated and matched three distinct iron-specific adducts: [M-2H+Fe]+, [M-H+Fe]2+, and [2M-2H+Fe]+.

RT Stability: All 41 siderophores exhibited a coefficient of variation (CV) for retention time below 2%, highlighting chromatographic consistency.

Visual Validation: We performed a visual inspection of all extracted ion chromatograms (EICs), accepting only those with sharp Gaussian peaks.
We revised the manuscript to consistently use the term "putatively identified" or "annotated" to remain within conservative scientific boundaries.

- **Reviewer Comment:** The manuscript concludes that marine sponges represent reservoirs of siderophores. I find this conclusion so premature since the compounds were not structurally confirmed, and the microbial source was not verified. In addition, no gene clusters or microbial isolation were performed

Response: We acknowledge that our identification is at the putative annotation level (MSI Level 2) and that we did not perform microbial isolation or gene cluster analysis. However, we respectfully argue that our conclusion is not premature when considered within the context of the existing literature. The term “reservoir” in our context means a source or holder of siderophore diversity. It is well established in the literature that marine sponges harbor dense and diverse microbial communities, including bacterial taxa known to produce siderophores (e.g., Pseudomonas, Streptomyces, and various Proteobacteria) (Thomas et al., 2016, ref. [46]). The siderophores we annotated (e.g., Ferricrocin, Aeruginic acid, Madurastatin) are known products of bacterial and fungal genera commonly found in sponge microbiomes. Our study was designed as an approach, and the detection of known siderophore signatures in sponge extracts provides indirect but compelling evidence for their microbial origin within the holobiont. However, we soften the language in the Conclusions section to reflect the putative nature of the annotations and the need for future studies to confirm the microbial sources through metagenomic and/or cultivation-based approaches.

- **Reviewer Comment**: The identified siderophores are not well discussed, and this does not bring out the significance of the study.

Response. We acknowledge that the ecological significance of the individual siderophores could be discussed more thoroughly, and we expanded the Discussion section accordingly in the revised manuscript

- **Reviewer Comment:** Long sentences (e.g., the "amalgamation" sentence), typos ("smaple"), and inconsistent reference style.

Response: We appreciate the attention to those details. We simplified the complex sentences cited by the reviewer to improve readability (e.g., replacing "The amalgamation of these analytical tools engenders..." with "Integrating these tools creates an efficient workflow..."). All typos, including "smaple" and missing italics for species names, were corrected, and the reference section was strictly formatted to PLOS One requirements.

We believe these clarifications, supported by the data already present in our work, address the reviewer's concerns and demonstrate the validity of our strategy for siderophore putative annotation. All changes can be observed in the revised manuscript. Likewise, the revised manuscript was suited for the PLOS ONE requirements.

Sincerely,

The Authors of PONE-S-26-08981
