# Supplementary material for: Siderophore screening in marine sponge extracts using LC-HRMS and an R-based metabolomics workflow
Source: PLoS One. 2026 Jun 17;21(6):e0343544. doi: 10.1371/journal.pone.0343544 (PMC13274820; doi:10.1371/journal.pone.0343544)
Supplement: Attachment — Submitted filename: Detailed Reviewers Report_Plos One Journal.doc [file pone.0343544.s003.doc]

**Detailed Reviewer’s Report**

**Title: Siderophore identification in microorganisms associated with marine sponges by LC-HRMS and a data analytic approach in R**

**General Comments**

The manuscript presents a computational workflow for detecting siderophores in marine sponge samples using LC-HRMS data and R-based metabolomics tools. The research work aimed to demonstrate that use of XCMS, MetaboAnnotation, and a curated siderophore database (SIDERITE) to identify siderophores directly from environmental extracts without microbial isolation.

The approach is okay and good for environmental metabolomics and natural product discovery. However, there are major issues that need to be addressed to improve the manuscript i.e. There is insufficient methodological detail, poor justification of experimental design, unclarity in sample preparation and replication and overinterpretation of tentative identifications. Therefore, in my assessment the current manuscript requires major revisions before it can be considered for publication in PLOS One Journal. Below are the section-by-section comments.

**Title:**

The title indicates the key area of study (siderophores), sample source (marine sponge–associated microorganisms), analytical technique (LC-HRMS), and computational platform (R). This reflects the methodological focus of the study.

**Major issues in the title**

- The title is not clear and it is misleading i.e. The title lacks clarity and is poorly structured.
- There is also ambiguity on the biological source i.e. the title states “microorganisms associated with marine sponges” However, the study analyses sponge extracts, not isolated microorganisms. This creates ambiguity that needs to be clarified by the authors. I would suggest the following title consideration for improvement:

(i) Siderophore screening in marine sponge extracts using LC-HRMS and an R-based metabolomics workflow.
(ii) LC-HRMS screening of siderophores in marine sponge extracts using an R-based metabolomics workflow.

**Introduction section**

- In the introduction section, the authors should have discussed other methodology that are used to identify and quantify the siderophores. The strength and weakness of such methods should have been highlighted.
- There are several statements that have not been supported with literature (The authors should provide relevant citations for these statements).

**Methodology section**

**Major concerns**

- Unclear sample collection and experimental Design. The sample collection procedure is insufficiently described, making it difficult to evaluate reproducibility. For instance, the manuscript indicates that samples of three sponge species were collected and 2 g of tissue were taken in triplicate, resulting in n = 36 samples, but the calculation of this sample number is not explained.

It is unclear whether the 36 samples represent biological replicates, technical replicates, or both. The sampling details (such as depth, site coordinates, collection method, and permission to undertake the study of marine sponges) are not described.

- The sample preservation procedure (such as immediate freezing, ethanol storage, transport conditions) is missing.

The authors should clarify how the sponges’ samples were collected, and identified. How were the samples preserved and processed?

- Importantly, the authors should address how the 36 total samples were derived!!
- The experiment includes iron supplementation with 200 μmol L⁻¹ Iron (III) ammonium citrate (FAS) prior to LC-MS analysis. But the authors did not justify the use of this concentration??? This has to be clarified. The use of the concentration appears arbitrary and no reference is provided for the selected iron concentration.

The authors should have indicated how iron supplementation may affect ionization efficiency and the biological induction of siderophores.

- The extraction protocol contains some inconsistencies. It is sated that: Three ethanol extractions (15 mL each) were performed and the supernatants were combined to give 45 mL of extract. The concern here is that Solvent loss typically occurs during extraction and centrifugation. So, it is unrealistic to recover exactly the same volume (45 mL) after three extractions.
- In addition, the mass of dried extract obtained is not reported and the final concentration of the extract prior to LC-MS injection is unclear.

The authors must indicate the average recovered solvent volume, dry extract yield (mg), the concentration of extract (mg/mL) and the exact mass injected into LC-MS. Such information is important for evaluating analytical sensitivity and reproducibility.

- Again, the manuscript does not clearly indicate whether or not the extraction procedures, and LC-MS injections, etc were replicated. For instance, the workflow mentions 36 LC-HRMS files, however, the relationship between samples, replicates, and treatments remains unclear.

**Metabolite Identification**

**Major concers**

- The study indicates that 41 siderophores were identified directly from sponge extracts. However, identification is based solely on accurate mass matching, adduct detection and retention time consistency. The major issue here is that mass matching alone cannot confirm metabolite identity. According to metabolomics standards (Metabolomics Standards Initiative). There are levels of identification that include: Level 1 that is confirmed with authentic standard; Level 2 that is putatively annotated, and Level 3, which is putatively characterized compound class.
- The authors should therefore, replace the words “identified siderophores” with “putatively annotated siderophores’’. They should also acknowledge limitations of database matching and explain the possible false positives due to isobaric compounds.

**Ecological Implications**

**Major concerns**

- The manuscript concludes that marine sponges represent reservoirs of siderophores. I find this conclusion so premature since the compounds were not structurally confirmed and the microbial source was not verified. In addition, no gene clusters or microbial isolation were performed.
- The identified siderophores are not well discussed and this does not bring out the significance of the study? Why was the identification of the siderophores important if their results are not well discussed. i.e. the authors should have discussed the ecological significance of the identified siderophores and why they are crucial in the symbiotic existence of sponges and associated microorganisms.

**Minor concerns**

- The manuscript also contains many long and unnecessarily complex sentences. For instance, from discussion: “The amalgamation of these analytical tools engenders a thorough and efficient workflow…” This type of language reduces readability. The authors should make use of simpler, more concise scientific language.
- There are missing references in some sections, particularly in sample description and the Iron supplementation rationale.
- There some grammatical errors including typos too: “smaple” instead of “sample”, Inconsistent use of italics for species names, inconsistent use of citation styles, and long and poorly constructed sentences especially in the discussion section. For example, ‘Lehner et al. 2013 conducted a comprehensive investigation that led to the identification and subsequent detection of 18 distinct iron chelators produced as a result of the laboratory cultivation of a collection comprising 10 wild-type strains of the *Trichoderma* genus [27]’; ‘Boiteau et al. 2019 identified 9 siderophores in culturing microorganisms from soil samples and reported the siderophore Schizokinen, also found in our screening [29].’’

Such sentences are not only long, but also unclear!!! The authors should revise such sentences or make use of short, clear and succinct sentences to improve communications.

**References:**

- There is no consistence in the reference style use. The authors should revise the reference section and follow the reference format requirement of PLOS One journal.
