# Supplementary material for: Siderophore screening in marine sponge extracts using LC-HRMS and an R-based metabolomics workflow
Source: PLoS One. 2026 Jun 17;21(6):e0343544. doi: 10.1371/journal.pone.0343544 (PMC13274820; doi:10.1371/journal.pone.0343544)
Supplement: Peer review history

# Peer review: Decision Letter 0

**DOI:** 10.1371/journal.pone.0343544.r001

**Authors:** Rachid Bouharroud

6 Apr 2026

PONE-D-26-06824

Siderophore identification in microorganisms associated with marine sponges by LC-HRMS and a data analytic approach in R.

PLOS One

Dear Dr. Arenas,

Thank you for submitting your manuscript to PLOS ONE. After careful consideration, we feel that it has merit but does not fully meet PLOS ONE’s publication criteria as it currently stands. Therefore, we invite you to submit a revised version of the manuscript that addresses the points raised during the review process.

Please submit your revised manuscript by May 21 2026 11:59PM. If you will need more time than this to complete your revisions, please reply to this message or contact the journal office at plosone@plos.org. When you're ready to submit your revision, log on to https://www.editorialmanager.com/pone/ and select the 'Submissions Needing Revision' folder to locate your manuscript file.

Please include the following items when submitting your revised manuscript:

- A letter that responds to each point raised by the academic editor and reviewer(s). You should upload this letter as a separate file labeled 'Response to Reviewers'.
- A marked-up copy of your manuscript that highlights changes made to the original version. You should upload this as a separate file labeled 'Revised Manuscript with Track Changes'.
- An unmarked version of your revised paper without tracked changes. You should upload this as a separate file labeled 'Manuscript'.

If you would like to make changes to your financial disclosure, please include your updated statement in your cover letter. Guidelines for resubmitting your figure files are available below the reviewer comments at the end of this letter.

If applicable, we recommend that you deposit your laboratory protocols in protocols.io to enhance the reproducibility of your results. Protocols.io assigns your protocol its own identifier (DOI) so that it can be cited independently in the future. For instructions see: https://journals.plos.org/plosone/s/submission-guidelines#loc-laboratory-protocols. Additionally, PLOS ONE offers an option for publishing peer-reviewed Lab Protocol articles, which describe protocols hosted on protocols.io. Read more information on sharing protocols at https://plos.org/protocols?utm_medium=editorial-email&utm_source=authorletters&utm_campaign=protocols.

As the corresponding author, your ORCID iD is verified in the submission system and will appear in the published article. PLOS supports the use of ORCID, and we encourage all coauthors to register for an ORCID iD and use it as well. Please encourage your coauthors to verify their ORCID iD within the submission system before final acceptance, as unverified ORCID iDs will not appear in the published article. *Only*  the individual author can complete the verification step; PLOS staff *cannot*  verify ORCID iDs on behalf of authors.

We look forward to receiving your revised manuscript.

Kind regards,

Rachid Bouharroud

Academic Editor

PLOS One

Journal Requirements:

When submitting your revision, we need you to address these additional requirements.

1.Please ensure that your manuscript meets PLOS ONE's style requirements, including those for file naming. The PLOS ONE style templates can be found at

https://journals.plos.org/plosone/s/file?id=wjVg/PLOSOne_formatting_sample_main_body.pdf and

https://journals.plos.org/plosone/s/file?id=ba62/PLOSOne_formatting_sample_title_authors_affiliations.pdf

2. In your Methods section, please provide additional information regarding the permits you obtained for the work. Please ensure you have included the full name of the authority that approved the field site access and, if no permits were required, a brief statement explaining why.

3. Please note that funding information should not appear in any section or other areas of your manuscript. We will only publish funding information present in the Funding Statement section of the online submission form. Please remove any funding-related text from the manuscript.

4. When completing the data availability statement of the submission form, you indicated that you will make your data available on acceptance. We strongly recommend all authors decide on a data sharing plan before acceptance, as the process can be lengthy and hold up publication timelines. Please note that, though access restrictions are acceptable now, your entire data will need to be made freely accessible if your manuscript is accepted for publication. This policy applies to all data except where public deposition would breach compliance with the protocol approved by your research ethics board. If you are unable to adhere to our open data policy, please kindly revise your statement to explain your reasoning and we will seek the editor's input on an exemption. Please be assured that, once you have provided your new statement, the assessment of your exemption will not hold up the peer review process.

5. Please include captions for your Supporting Information files at the end of your manuscript, and update any in-text citations to match accordingly. Please see our Supporting Information guidelines for more information: http://journals.plos.org/plosone/s/supporting-information.

6. If the reviewer comments include a recommendation to cite specific previously published works, please review and evaluate these publications to determine whether they are relevant and should be cited. There is no requirement to cite these works unless the editor has indicated otherwise.

7. Please review your reference list to ensure that it is complete and correct. If you have cited papers that have been retracted, please include the rationale for doing so in the manuscript text, or remove these references and replace them with relevant current references. Any changes to the reference list should be mentioned in the rebuttal letter that accompanies your revised manuscript. If you need to cite a retracted article, indicate the article’s retracted status in the References list and also include a citation and full reference for the retraction notice.

[Note: HTML markup is below. Please do not edit.]

Reviewers' comments:

Reviewer's Responses to Questions

**Comments to the Author**

1. Is the manuscript technically sound, and do the data support the conclusions?

The manuscript must describe a technically sound piece of scientific research with data that supports the conclusions. Experiments must have been conducted rigorously, with appropriate controls, replication, and sample sizes. The conclusions must be drawn appropriately based on the data presented.

Reviewer #1: Yes

Reviewer #2: No

**********

2. Has the statistical analysis been performed appropriately and rigorously?

Reviewer #1: Yes

Reviewer #2: Yes

**********

3. Have the authors made all data underlying the findings in their manuscript fully available?

The PLOS Data policy requires authors to make all data underlying the findings described in their manuscript fully available without restriction, with rare exception (please refer to the Data Availability Statement in the manuscript PDF file). The data should be provided as part of the manuscript or its supporting information, or deposited to a public repository. For example, in addition to summary statistics, the data points behind means, medians and variance measures should be available. If there are restrictions on publicly sharing data—e.g. participant privacy or use of data from a third party—those must be specified.

Reviewer #1: Yes

Reviewer #2: Yes

**********

4. Is the manuscript presented in an intelligible fashion and written in standard English?

PLOS ONE does not copyedit accepted manuscripts, so the language in submitted articles must be clear, correct, and unambiguous. Any typographical or grammatical errors should be corrected at revision, so please note any specific errors here.

Reviewer #1: Yes

Reviewer #2: No

**********

5. Review Comments to the Author

Please use the space provided to explain your answers to the questions above. You may also include additional comments for the author, including concerns about dual publication, research ethics, or publication ethics. (Please upload your review as an attachment if it exceeds 20,000 characters)

Reviewer #1: The topic is highly relevant, the manuscript meets the standards of scientific writing, the methodology is original and well conceived, and the results are rich and insightful

I have no additional comments.

Reviewer #2: The manuscript cannot be considered technically sound in its current form, and the data do not adequately support the conclusions. The study lacks sufficient transparency and detail to be reproducible, which is a core requirement for technical soundness.

The manuscript has major methodological weaknesses that undermine reproducibility and rigor:

Sampling design is unclear i.e. the derivation of n = 36 samples is not explained. There is no distinction between biological vs. technical replicates. In addition, there is missing metadata (sampling depth, coordinates, collection method, and permits) and the sample handling is inadequately described

The experimental treatment lacks justification, i.e. the use of 200 μmol L⁻¹ iron (III) ammonium citrate is arbitrary and unreferenced.

Also, the extraction protocol is inconsistent. For instance, reporting an exact recovery of 45 mL after 3 × 15 mL extractions is unrealistic. There is also missing key quantitative details: i.e. actual recovered volume , dry extract yield (mg), Final concentration (mg/mL), and injection mass for LC-MS

The replication and analytical workflow unclear. For example, the relationship between 36 LC-HRMS files, samples, and replicates is not defined. Thus, the study lacks clarity on technical reproducibility.

The claim of “41 identified siderophores” is overstated since the identification is based only on: Accurate mass, adducts, and retention time.

Therefore, the compounds should be described as “putatively annotated siderophores”, not definitively identified. There is a high risk of false positives, especially from isobaric compounds.

The conclusions are not sufficiently supported. i.e. the claim that ‘Marine sponges are reservoirs of siderophores’ is not validated because the compounds are not structurally confirmed, there is no evidence of biosynthetic origin (host vs. microbes), and no genomic (BGCs) or microbial isolation data. There weak ecological interpretation with minimal discussion on the functional roles of siderophores on sponge–microbe symbiosis and their environmental relevance.

In addition, the manuscript is not consistently presented in an intelligible fashion and does not fully meet the standards of clear, formal scientific English, although the issues can be improved with careful revision.

The manuscript uses overly complex and verbose language, particularly in the discussion (e.g., phrases like “The amalgamation of these analytical tools engenders…”). Such words may reduces readability and cause difficulty in communication.

Scientific writing should prioritize clarity over sophistication, and many sentences would benefit from simplification.

There are also multiple examples of excessively long sentences, poorly constructed statements with unclear meaning. This may significantly affects the intelligibility of the manuscript, especially in the discussion section.

There are also grammar, typos, and formatting Issues. For instance, the presence of typographical errors (e.g., “smaple” instead of “sample”), and inconsistent formatting, including italics for species names and citation styles. All these issues indicate insufficient proofreading and reduce the overall professionalism of the manuscript.

There also missing references in key sections such as sample description, the Iron supplementation rationale. Etc. This not only affects scientific rigor but also disrupts the logical flow and credibility of the text.

**********

6. PLOS authors have the option to publish the peer review history of their article (what does this mean?). If published, this will include your full peer review and any attached files.

If you choose “no”, your identity will remain anonymous but your review may still be made public.

**Do you want your identity to be public for this peer review?**  For information about this choice, including consent withdrawal, please see our Privacy Policy.

Reviewer #1: No

Reviewer #2: **Yes:**  Huxley Makonde

**********

[NOTE: If reviewer comments were submitted as an attachment file, they will be attached to this email and accessible via the submission site. Please log into your account, locate the manuscript record, and check for the action link "View Attachments". If this link does not appear, there are no attachment files.]

To ensure your figures meet our technical requirements, please review our figure guidelines: https://journals.plos.org/plosone/s/figures

You may also use PLOS’s free figure tool, NAAS, to help you prepare publication quality figures: https://journals.plos.org/plosone/s/figures#loc-tools-for-figure-preparation.

NAAS will assess whether your figures meet our technical requirements by comparing each figure against our figure specifications.

Attachment Submitted filename: Detailed Reviewers Report_Plos One Journal.doc

# Author comment: Author response to Decision Letter 1

**DOI:** 10.1371/journal.pone.0343544.r002

6 May 2026

Dear Editor and reviewers,

On behalf of all authors, we would like to thank you and the reviewers for the

constructive feedback and the opportunity to revise our manuscript, "Siderophore

identification in microorganisms associated with marine sponges by LC-HRMS and a

data analytic approach in R" (PONE-S-26-08981).

Below, we provide a point-by-point response to the reviewer’s comments.

- Reviewer Comment: The title is misleading as the study analyzes sponge

extracts, not isolated microorganisms. Suggests: "Siderophore screening in

marine sponge extracts..."

Response: We acknowledge the reviewer's concern regarding the biological source.

Our study utilizes a metabolomics approach, aiming to capture the Siderophore

landscape of the sponge holobiont (the host and its complex microbial community).

To resolve any ambiguity, we change the title to: "Siderophore screening in marine

sponge extracts using LC-HRMS and an R-based metabolomics workflow."

- Reviewer Comment: In the introduction section, the authors should have

discussed other methodologies that are used to identify and quantify the

siderophores. The strengths and weaknesses of such methods should have

been highlighted.

Response: We added the two paragraphs below in the introduction section

regarding other methods to identify Siderophores.

Several methodologies have been developed for the detection, identification, and

quantification of siderophores, each with distinct strengths and limitations. The

Chrome Azurol S (CAS) assay is a widely used colorimetric method that detects

siderophore-mediated iron chelation through a characteristic color change, providing

a simple and rapid screening tool for siderophore production. However, the CAS

assay is non-specific, as it cannot distinguish between different siderophore types or

identify individual structures, and it is subject to interference from other iron-chelating

compounds in complex matrices. Bioassays employing siderophore-utilizing indicator

strains offer biological activity information and can reveal functional siderophore

diversity, yet these approaches are inherently cultivation-dependent and

low-throughput, limiting their applicability to environmental samples with unculturable

microbiomes. Nuclear magnetic resonance (NMR) spectroscopy provides definitive

structural elucidation of purified siderophores; however, NMR requires pure

compounds in milligram quantities and is impractical for the analysis of complex

environmental extracts where siderophores occur at low concentrations amid

abundant co-extracted metabolites. Liquid chromatography coupled with tandem

mass spectrometry (LC-MS/MS) and molecular networking platforms such as the

Global Natural Products Social Molecular Networking (GNPS) enable structural

annotation through fragmentation pattern analysis and spectral library matching, but

these approaches depend heavily on the availability of well-curated MS/MS spectral

libraries, which remain limited for many siderophore classes.

“Compared to the methods described above, LC-HRMS with accurate mass

matching offers a complementary balance of sensitivity, throughput, and broad

coverage for initial screening of complex environmental samples, while

acknowledging that it does not replace definitive structural confirmation by NMR or

MS/MS-based approaches.”

- Reviewer Comment: Several statements have not been supported with

literature (the authors should provide relevant citations for these statements).

Response: We carefully reviewed the entire introduction section to ensure that

relevant citations properly support all statements. In the revised manuscript, we

added new references for any uncited claims.

- Reviewer Comment: Unclear sample collection and experimental Design.

Response: We clarified in the revised text all issues regarding sample acquisition

and the inconsistency in the methodology. The following paragraph was replaced in

the methodology section “Samples”.

“Samples were collected from three sponge species: D. reticulatum, A. fulva, and A.

viridis, along the São Paulo coast, Brazil, at depths of 3–8 m, by SCUBA diving

(ICMBio permit #0289170220250814). Three independent individuals per species

were sampled as biological replicates. From each individual, two separate 2-g

portions of body tissue were excised from two different anatomical regions, serving

as technical replicates, for a total of 18 samples (3 species × 3 individuals × 2 tissue

portions). Immediately after collection, samples were flash-frozen in liquid nitrogen

on board, transported on dry ice, and stored at −80 °C until extraction.

Following methanol extraction of the 18 samples, the resulting extracts were each

split into two aliquots: one supplemented with 200 μmol L⁻¹ Ferrous Ammonium

Sulfate (FAS) (final concentration 4 μmol L⁻¹) as an iron source to promote the

formation of iron adducts [22]; the control aliquot was supplemented with 2 μL of

ultrapure water. This yielded a total of 36 samples for LC-HRMS injection.”

- Reviewer Comment: Why was 200 µmol/L used? Why Iron(III) Ammonium

Citrate (FAS)?

Response: First, we corrected the substance used: Iron(III) ammonium citrate

(FAS), which is not the compound used in the chelation stimulation protocol. The

substance used was Ferrous Ammonium Sulfate (FAS). The concentration of 200

µmol/L was selected based on established siderophore induction and detection

in-house protocols in our lab, providing sufficient iron to saturate available ligands

without inducing toxicity or massive precipitation that could interfere with LC-HRMS

analysis. Ferrous Ammonium Sulfate (FAS) was chosen for its high solubility in both

water and organic mixtures, ensuring consistent iron availability. The fact that

supplementation did not significantly alter detection suggests that the identified

siderophores were already present as iron-complexes (ferri-siderophores) or were

constitutively produced within the sponge environment. In this reference, “Batko, I.

Z., Flannagan, R. S., Guariglia-Oropeza, V., Sheldon, J. R., & Heinrichs, D. E.

(2021). Heme-Dependent Siderophore Utilization Promotes Iron-Restricted Growth

of the Staphylococcus aureus hemB Small-Colony Variant. Journal of bacteriology,

203(24), e0045821. https://doi.org/10.1128/JB.00458-21” [22]. The authors use a

concentration of 10 μM of FAS to complement the growth of a Staphylococcus

aureus hemB Small-Colony Variant, which is a bacterium defective in growing in

media with iron deficiency. Adding FAS restores bacterial growth. The final

concentration of FAS used in our experiment was 4 μmol L⁻¹ since we added 2 μL of

200 μmol L⁻¹ Ferrous Ammonium Sulfate (FAS) to 98 μL of extract before LC-HRMS

injection. All those quantities and concentrations were clarified in the revised

manuscript.

- Reviewer Comment: The mass of dried extract obtained is not reported, and

the final concentration of the extract before LC-MS injection is unclear.

Response: We acknowledge that this information is important for analytical

reproducibility and will add it to the revised manuscript. While we did not record

individual dry weights for each of the 18 biological samples (as this was not a

primary objective of the study), we can provide the following details: the final

reconstitution volume was 250 μL of dry methanol, and the injection volume for

LC-HRMS was 5 μL per run. Based on our experience with similar marine sponge

ethanol extracts processed through the same fractionation workflow, the typical dry

extract yield from 2 g of wet sponge tissue after chloroform partition is in the range of

5–20 mg, corresponding to a final concentration of approximately 5 mg/mL in 250 μL

methanol. We will add these estimates to the revised manuscript. We will also note

that the injection of 5 μL at these concentrations is consistent with standard

LC-HRMS practices for natural product screening.

- Reviewer Comment: Claims of identifying specific compounds (Ferricrocin,

etc.) are overinterpreted without MS/MS or standards.

Response: We agree that in untargeted metabolomics, these represent putative

identifications (Level 2/3 according to the Metabolomics Standards Initiative).

However, we defend our current results based on our multi-orthogonal validation

pipeline:

Mass Accuracy: All reported features had a mass error ppm < 3.

Adduct Matching: We didn't rely on a single mass; we calculated and matched three

distinct iron-specific adducts: [M-2H+Fe]+, [M-H+Fe]2+, and [2M-2H+Fe]+.

RT Stability: All 41 siderophores exhibited a coefficient of variation (CV) for retention

time below 2%, highlighting chromatographic consistency.

Visual Validation: We performed a visual inspection of all extracted ion

chromatograms (EICs), accepting only those with sharp Gaussian peaks.

We revised the manuscript to consistently use the term "putatively identified" or

"annotated" to remain within conservative scientific boundaries.

- Reviewer Comment: The manuscript concludes that marine sponges

represent reservoirs of siderophores. I find this conclusion so premature since

the compounds were not structurally confirmed, and the microbial source was

not verified. In addition, no gene clusters or microbial isolation were

performed

Response: We acknowledge that our identification is at the putative annotation level

(MSI Level 2) and that we did not perform microbial isolation or gene cluster

analysis. However, we respectfully argue that our conclusion is not premature when

considered within the context of the existing literature. The term “reservoir” in our

context means a source or holder of siderophore diversity. It is well established in the

literature that marine sponges harbor dense and diverse microbial communities,

including bacterial taxa known to produce siderophores (e.g., Pseudomonas,

Streptomyces, and various Proteobacteria) (Thomas et al., 2016, ref. [46]). The

siderophores we annotated (e.g., Ferricrocin, Aeruginic acid, Madurastatin) are

known products of bacterial and fungal genera commonly found in sponge

microbiomes. Our study was designed as an approach, and the detection of known

siderophore signatures in sponge extracts provides indirect but compelling evidence

for their microbial origin within the holobiont. However, we soften the language in the

Conclusions section to reflect the putative nature of the annotations and the need for

future studies to confirm the microbial sources through metagenomic and/or

cultivation-based approaches.

- Reviewer Comment: The identified siderophores are not well discussed, and

this does not bring out the significance of the study.

Response. We acknowledge that the ecological significance of the individual

siderophores could be discussed more thoroughly, and we expanded the Discussion

section accordingly in the revised manuscript

- Reviewer Comment: Long sentences (e.g., the "amalgamation" sentence),

typos ("smaple"), and inconsistent reference style.

Response: We appreciate the attention to those details. We simplified the complex

sentences cited by the reviewer to improve readability (e.g., replacing "The

amalgamation of these analytical tools engenders..." with "Integrating these tools

creates an efficient workflow..."). All typos, including "smaple" and missing italics for

species names, were corrected, and the reference section was strictly formatted to

PLOS One requirements.

We believe these clarifications, supported by the data already present in our work,

address the reviewer's concerns and demonstrate the validity of our strategy for

siderophore putative annotation. All changes can be observed in the revised

manuscript. Likewise, the revised manuscript was suited for the PLOS ONE

requirements.

Sincerely,

The Authors of PONE-S-26-08981

Attachment Submitted filename: Response to Reviewers.pdf

# Editor report: Decision Letter 1

**DOI:** 10.1371/journal.pone.0343544.r003

**Authors:** Rachid Bouharroud, Rachid Bouharroud

12 May 2026

<p>Siderophore screening in marine sponge extracts using LC-HRMS and an R-based metabolomics workflow.

PONE-D-26-06824R1

Dear Dr. Arenas,

We’re pleased to inform you that your manuscript has been judged scientifically suitable for publication and will be formally accepted for publication once it meets all outstanding technical requirements.

Within one week, you’ll receive an e-mail detailing the required amendments. When these have been addressed, you’ll receive a formal acceptance letter and your manuscript will be scheduled for publication.

An invoice will be generated when your article is formally accepted. Please note, if your institution has a publishing partnership with PLOS and your article meets the relevant criteria, all or part of your publication costs will be covered. Please make sure your user information is up-to-date by logging into Editorial Manager at Editorial Manager® and clicking the ‘Update My Information' link at the top of the page. For questions related to billing, please contact billing support.

If your institution or institutions have a press office, please notify them about your upcoming paper to help maximize its impact. If they’ll be preparing press materials, please inform our press team as soon as possible -- no later than 48 hours after receiving the formal acceptance. Your manuscript will remain under strict press embargo until 2 pm Eastern Time on the date of publication. For more information, please contact onepress@plos.org.

Kind regards,

Rachid Bouharroud

Academic Editor

PLOS One

Additional Editor Comments (optional):

Reviewers' comments:

# Editor report: Acceptance letter

**DOI:** 10.1371/journal.pone.0343544.r004

**Authors:** Rachid Bouharroud, Rachid Bouharroud

PONE-D-26-06824R1

PLOS One

Dear Dr. Arenas-Soto,

I'm pleased to inform you that your manuscript has been deemed suitable for publication in PLOS One. Congratulations! Your manuscript is now being handed over to our production team.

At this stage, our production department will prepare your paper for publication. This includes ensuring the following:

* All references, tables, and figures are properly cited

* All relevant supporting information is included in the manuscript submission,

* There are no issues that prevent the paper from being properly typeset

You will receive further instructions from the production team, including instructions on how to review your proof when it is ready. Please keep in mind that we are working through a large volume of accepted articles, so please give us a few days to review your paper and let you know the next and final steps.

Lastly, if your institution or institutions have a press office, please let them know about your upcoming paper now to help maximize its impact. If they'll be preparing press materials, please inform our press team within the next 48 hours. Your manuscript will remain under strict press embargo until 2 pm Eastern Time on the date of publication. For more information, please contact onepress@plos.org.

You will receive an invoice from PLOS for your publication fee after your manuscript has reached the completed accept phase. If you receive an email requesting payment before acceptance or for any other service, this may be a phishing scheme. Learn how to identify phishing emails and protect your accounts at https://explore.plos.org/phishing.

If we can help with anything else, please email us at customercare@plos.org.

Thank you for submitting your work to PLOS ONE and supporting open access.

Kind regards,

PLOS ONE Editorial Office Staff

on behalf of

Dr. Rachid Bouharroud

Academic Editor

PLOS One
